# Ibrutinib Does Not Impact CCR7-Mediated Homeostatic Migration in T-Cells from Chronic Lymphocytic Leukemia Patients

**DOI:** 10.3390/cancers14112729

**Published:** 2022-05-31

**Authors:** Tamara Mateu-Albero, Ana Marcos-Jimenez, Stefanie Wissmann, Javier Loscertales, Fernando Terrón, Jens V. Stein, Cecilia Muñoz-Calleja, Carlos Cuesta-Mateos

**Affiliations:** 1 Immunology Department, Hospital Universitario de La Princesa, IIS-IP, 28006 Madrid, Spain; tamaltma@gmail.com (T.M.-A.); amarcos@salud.madrid.org (A.M.-J.); cmunozc@salud.madrid.org (C.M.-C.); 2 Department of Oncology, Microbiology and Immunology, University of Fribourg, CH-1700 Freiburg, Switzerland; stefanie.wissmann@unifr.ch (S.W.); jens.stein@unifr.ch (J.V.S.); 3 Hematology Department, Hospital Universitario de La Princesa, IIS-IP, 28006 Madrid, Spain; javier.loscertales@salud.madrid.org; 4 IMMED S.L., Immunological and Medicinal Products, C/Velázquez 57, 6º derecha, 28001 Madrid, Spain; fterron@biolty.com; 5 Catapult Therapeutics, 8243 RC Lelystad, The Netherlands; 6 School of Medicine, Universidad Autónoma de Madrid, 28029 Madrid, Spain

**Keywords:** T-cell, CCR7, CLL, ibrutinib, lymph node, migration

## Abstract

**Simple Summary:**

Bruton’s tyrosine kinase inhibitor ibrutinib restores T-cell immunity and increases circulating T-cell numbers in chronic lymphocytic leukemia (CLL) patients. Recent evidence suggests T-cell enhanced expansion, rather than increased egress from secondary lymphoid organs (SLO), as a root cause for ibrutinib-induced lymphocytosis. However, this physiological change might reflect, as well, a forced retention in PB derived of impaired migration to SLO. With the aim to investigate the impact of ibrutinib on CCR7-mediated homeostasis in CLL T-cells, we have documented receptor expression in a large cohort of ibrutinib-treated patients, and performed different in vivo and in vitro migration models. We show that ibrutinib has no effect on CCR7 expression or receptor-mediated T-cell chemotaxis, homing to SLO, and interstitial migration. Together, these results indicate that ibrutinib T-lymphocytosis is not caused by accumulation in the blood stream, and therefore back the T-cell expansion as the most plausible cause.

**Abstract:**

Bruton’s tyrosine kinase inhibitor ibrutinib has significantly changed treatment landscape in chronic lymphocytic leukemia (CLL). Growing evidence supports ibrutinib to work beyond the effect on tumor cells by means of, for example, restoring functionality of the T-cell compartment and increasing circulating T-cell numbers. Recent evidence suggests T-cell enhanced expansion, rather than increased egress from secondary lymphoid organs (SLO), as a root cause for ibrutinib-induced lymphocytosis. However, whether the latter physiological change is also a consequence of a forced retention in blood remains undisclosed. Since CCR7 is the main chemokine receptor taking over the homing of T-cells from peripheral compartments to lymph nodes and other SLO, we aimed to investigate the impact of ibrutinib on CCR7 functionality in T-cells. To this end, we documented receptor expression in T-cells from a large cohort of ibrutinib-treated CLL patients, and performed different in vivo and in vitro migration models. Overall, our data confirm that CCR7 expression or receptor-mediated migration in CLL T-cells is not affected by ibrutinib. Furthermore, it does not modulate CCR7-driven homing nor nodal interstitial migration. Together, our results support that ibrutinib-induced CLL T-cell accumulation in the blood stream is not derived from an impairment of CCR7-driven recirculation between the SLO and bloodstream, and therefore T-cell expansion is the most plausible cause.

## 1. Introduction

A hallmark of chronic lymphocytic leukemia (CLL) is the presence of a dysfunctional T-cell compartment [1,2]. Ibrutinib, the first-in-class oral inhibitor of the Bruton’s tyrosine kinase (BTK) to be approved, has achieved unprecedented clinical activity in CLL [3,4,5,6]. While first data supported irreversible antagonism of BTK in leukemic cells as the main mechanism of action of the drug [7,8,9,10,11], recent works confirm that a limited number of kinases can be inhibited at clinically achievable concentrations by ibrutinib as well (e.g., the interleukine-2-inducible kinase, ITK) [12]. As a result, ibrutinib restores CLL T-cells deregulation [12,13,14,15,16], eventually modulating towards anti-tumor T-cell responses [17,18]. Today, several immunomodulatory mechanisms of ibrutinib on CLL T-cell homeostasis have been uncovered [19]; however, there are still many of them that we are only beginning to understand. This is the case of ibrutinib-induced CLL T-cell lymphocytosis that might stem from either an impaired trafficking to secondary lymphoid organs (SLO), and more specifically to lymph nodes (LN), or an increased egress from these tissues. Long et al. recently discarded ibrutinib to potentiate CLL T-cells output flow in vivo [14], but its effect on LN or spleen ingress remains largely unknown. To gain insight into this topic, we focused on the homeostatic chemokine receptor CCR7 which, upon binding to its cognate ligands CCL19 and CCL21, controls the homing of certain immune cell subsets into T-cell specific regions within SLO and favors T-cell responses [20,21]. By means of in vivo and in vitro migration models, and by analyzing CCR7 expression in pan-T-cells from a large cohort of ibrutinib-treated CLL patients [22], herein we document the effect of ibrutinib on CCR7 expression and functionality in CLL T-cells.

## 2. Materials and Methods

### 2.1. Samples

Peripheral blood (PB) mononuclear cells (PBMC) were isolated from fresh samples. Patients signed an informed consent in accordance to the Declaration of Helsinki. Experimental work was approved by the Institutional review Board of Hospital de La Princesa. Three types of patient samples (n = 215) were analyzed: treatment-naïve patients (n = 144), patients on treatment (OT) with ibrutinib (n = 52), and ibrutinib relapsed/refractory patients (n = 19). The characteristics of samples used in this study have been disclosed before [22]. 

### 2.2. Flow Cytometry

Analysis of CCR7 expression was conducted, according to published protocols [22], in whole PB samples with a four-color panel of fluorochrome-labelled monoclonal antibody (mAb): CD19-APC/H7 (SJ25C1), CD3-FITC (SK7) and CD5-APC (L17F12) from BD Biosciences (San Jose, CA, USA), and CCR7-PE (clone 150503) from R&D Systems (Minneapolis, MN). A corresponding PE-conjugated isotype control (IC) was included (R&D Systems). Results are expressed both as a percentage of positive cells and relative median of fluorescence intensity (RMFI) of receptor expression compared to the IC [MFI(test)/MFI(control)].

### 2.3. Migration Assays

Chemotaxis towards CCL19 or CCL21 (1 µg/mL, PeproTech, Rocky Hills, NJ, USA) was assayed with Transwell inserts (5-µm diameter pore size, Corning-Costar, Tewksbury, MA, USA) following reported protocols [23,24]. If needed, cells were pre-exposed for 30 min to anti-CCR7 mAb CAP-100 (10 µg/mL, Catapult Therapeutics B.V, The Netherlands), or for three (or 24) hours to ibrutinib [0, 0.01, 0.1, 1, 10 µM] (obtained from La Princesa Pharmacy Service) within published dose ranges [10,11]. The proportion of migrating CD3^+^ pan-T-cells (% of input) was obtained with the formula: (nº cells counted in the lower well/nº cells loaded in the insert) × 100. 

For migration assays with murine lymphocytes, single cell suspensions obtained from peripheral LN and spleens were pretreated for 2–3 h with ibrutinib and allowed to migrate towards mouse recombinant CCL21 (1 µg/mL, PeproTech), in the presence of the inhibitor. The proportion of migrating T-cell subsets was determined by flow cytometry with a panel of mAbs: CD4-FITC (RM4-5, BioLegend, San Diego, CA, USA), CD8-PerCP (53-6.7, BioLegend), and CD45-R/B220 APC (RA3-6B2, BD Biosciences). Surface expression of CCR7 and adhesion molecules was assayed with CD62L-BV421 (MEL-14, BioLegend) or PE-conjugated mAbs: CCR7 (4B12, BioLegend), CD44 (IM7, BD Biosciences), very-late antigen-4 (VLA-4 or CD49d) (PS/2, Southern Biotech, Birmingham, AL, USA), lymphocyte function-associated antigen-1 (LFA-1 or CD11a or αL) (M17/4, BioLegend).

### 2.4. In Vivo Intranodal Migration: Two-Photon Microscopy (2PM)

In vivo models were conducted at the University of Bern and the University of Fribourg. Animal work was approved by the Cantonal Committees for Animal Experimentation and conducted according to federal guidelines. Murine T- and B-cells were isolated from LNs and spleens using Easy Sep T- or B-cell Isolation Kits (STEMCELL Technologies, Vancouver, Canada). Cells were stained with Cell Trackers CMTMR, CMAC or CFSE (ThermoFisher Scientific, Waltham, MA, USA), according to manufacturer’s instructions. Then, T- and B-cells were mixed 1:1 and ~5 million cells were adoptively transferred into 6–20 weeks old in-house-bred C57BL/6J recipients and allowed to recirculate for 48 h. On the following three days, mice were daily treated with i.p. 6 mg/kg ibrutinib or vehicle dimethyl sulfoxide (DMSO), as previously reported [25]. These time points correspond to the period when transient lymphocytosis is seen after ibrutinib treatment in a CLL mouse model [10,14]. The day of the experiment, 1 h after the administration of the drug, mice were anesthetized and the surgery was performed to expose the popliteal LN. In the following 2 h, 2PM images were taken as described [26,27]. Before image acquisition, high endothelial venules (HEVs) were labelled by i.v. injecting 10 µg of Alexa-Fluor 633-labeled Meca79 mAb. Z-stacks were acquired every 20 s during 20–30 min intervals, with and Olympus BX50WI fluorescence microscope equipped with a 20 × NA objective and a TrimScope 2PM system controlled by Imspector software (LaVision Biotec, Bielefeld, Germany). 3D-time lapse movies were analyzed using Imaris software (Bitplane, Belfast, UK). Cells attached to HEVs, or migrating through them, were discarded from the analysis, as their speed differs from that of cells migrating in the parenchyma.

### 2.5. In Vivo Homing

Lymphocytes were stained with 5µM CellTrace violet (CTV, Thermofisher Scientific) or 1 µM carboxyfluorescein succinimidyl sster (CFSE, Thermofisher Scientific), for 20 min at 37 °C. Cells were then pre-treated with 1µM ibrutinib (CFSE-stained) or DMSO/vehicle (CTV-stained) for 2 h at 37 °C and mixed in a 1:1 ratio in order to adoptively transfer a total of 10^7^ cells/mouse. After 1 h, PB, LN and spleens were collected and disaggregated by mechanical disruption. Cell suspensions (5 × 10^6^ cells) were stained with CD90.2-PerCP/Cy5.5 (53-2.1), CD4-PE/Cy7 (RM4-4), CD8-APC/Fire 750 (53-6.7) and B220-APC (RA3-6B2) (BioLegend). The Zombie Red™ Fixable Viability Kit (BioLegend) was used to exclude dead cells. Cells were acquired by flow cytometry using a BDLSR Fortessa (BD Biosciences) and the ratio ibrutinib/DMSO-treated B and T-cells was assessed using the FlowJo software (BD Bioscience). 

### 2.6. Statistical Analysis

Except as otherwise indicated, data are represented as measures of central tendency and dispersion (mean ± SD or SEM). For parametric variables, two sample *t*-test or one-way ANOVA were used. For heteroscedasticity, a Mann-Whitney-U or Kruskal-Wallis were used for unpaired samples whereas Wilcoxon-rank-sum or Friedman tests were used for unpaired samples. *p*-values were regarded as statistically significant according to the following criteria: *p* < 0.05 (*), *p* < 0.01 (**), *p* < 0.001 (***), or *p* < 0.0001 (****). 

## 3. Results

### 3.1. CCR7 Surface Expression Does Not Change in T-Cells from CLL Patients Treated with Ibrutinib

**Figure 1:**
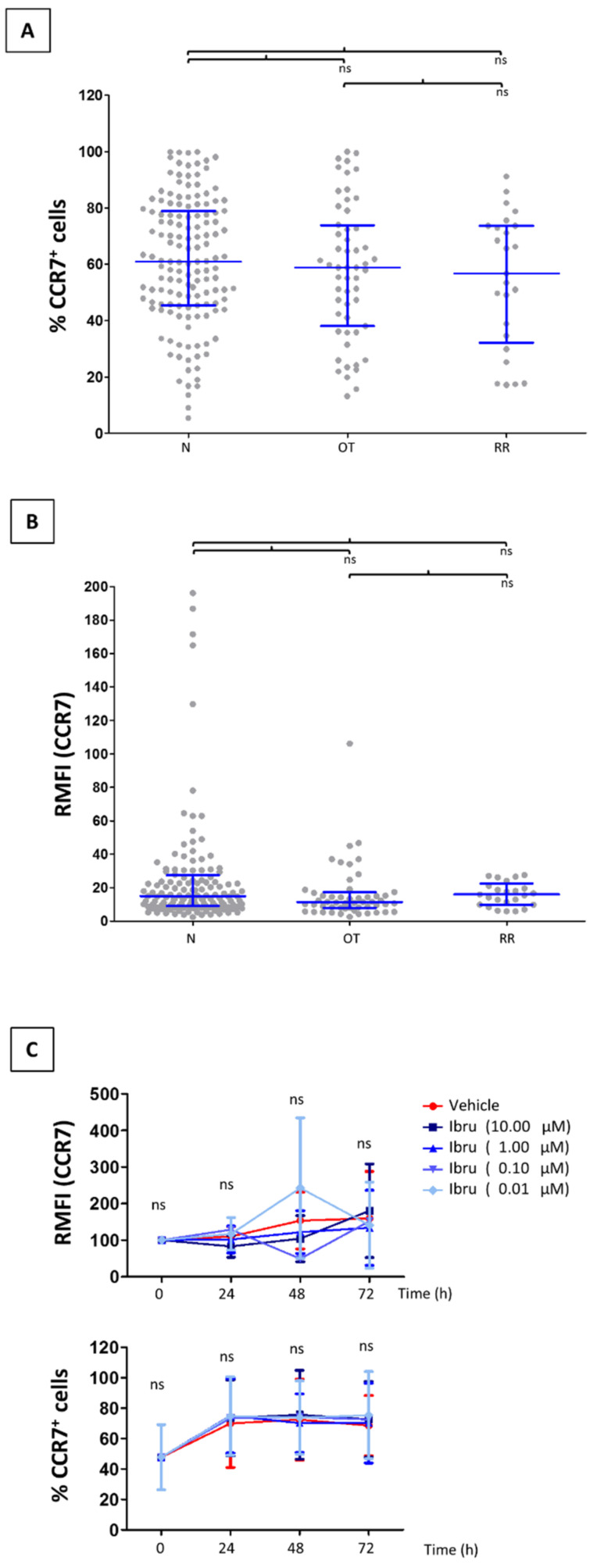
Ibrutinib does not modify CCR7 expression in chronic lymphocytic leukemia (CLL) T-cells from treated patients. (**A**,**B**) The expression of surface CCR7 was analyzed in terms of proportion of CLL T-cells expressing the receptor or of relative median fluorescence intensity (RMFI, relative to an irrelevant isotype control, arbitrary units). Expression was determined in PB samples from naïve patients (N, n = 144), patients on current treatment with ibrutinib (OT, n = 52), or patients with ibrutinib relapsed/refractory CLL (RR, n = 19). The graphs represent the median ± interquartile range. (**C**) CCR7 expression is maintained in CLL T-cells after in vitro incubation with ibrutinib. CLL T-cells from treatment-naïve patients (n = 7) were incubated for 72 h in the presence of ibrutinib at different final concentrations (0/DMSO; 0.01; 0.1; 1; 10 µM). At time 0, and every 24 h, CCR7 expression was determined by flow cytometry. The graphs show the proportion of CLL T-cells expressing CCR7, or the RMFI (arbitrary units). In all graphs: ns, not significant.

While CLL B-cells overexpress CCR7 [28], T-cells from treatment-naïve patients have comparable expression as healthy counterparts [29,30,31]. We and others have previously shown ibrutinib to cause a clear reduction of surface CCR7 levels in CLL B-cells that may eventually affect trafficking of malignant cells into the LN [22,32,33]. Since no study has addressed before whether a similar reduction takes place in CLL T-cells, we studied CCR7 expression in our CLL cohort [22]. We examined CCR7 expression in PB CD3^+^ pan-T-cells from patients on treatment with ibrutinib at the time of sampling and from patients who had developed relapsed/refractory disease, and compared it to cells obtained from naïve, untreated patients. The proportion of CCR7 expressing CD3^+^ T-cells was similar regardless current or previous treatment with ibrutinib (Figure 1A). In all groups, the patient-to-patient heterogeneity agreed with that seen in other CLL cohorts and healthy donor counterparts [29,30,31]. In terms of RMFI, CCR7 expression was not down-regulated in CLL T-cells from patients receiving ibrutinib or with relapsed/refractory disease, when compared to controls (Figure 1B). These results were further corroborated by means of in vitro approaches where T-cells from treatment-naïve CLL patients were incubated with different concentrations of ibrutinib (Figure 1C). Overall, these results confirmed that ibrutinib had no impact on the CCR7 surface expression of CLL T-cells.

### 3.2. Ibrutinib Does Not Modulate CCR7-Mediated In Vitro Migration of CLL T-Cells

**Figure 2:**
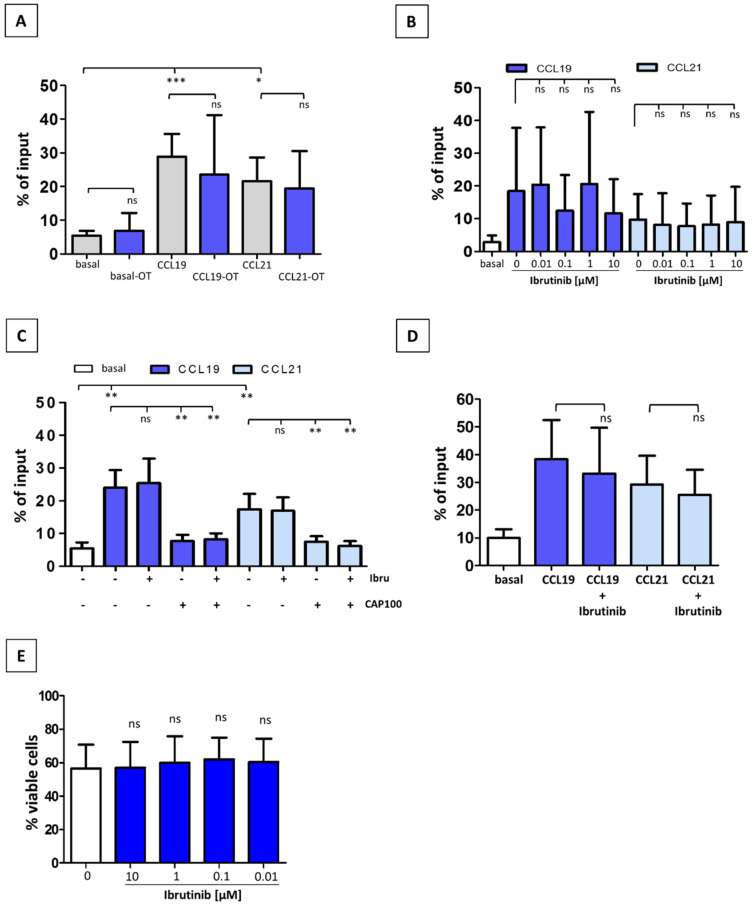
Ibrutinib has no effect on CCR7-driven migration in chronic lymphocytic leukemia (CLL) T-cells. (**A**) Analysis of the in vitro migration index (% of input) of T-cells obtained from naïve, untreated CLL patients (N; n = 7, basal, CCL19 and CCL21) versus T-cells from patients treated with ibrutinib (OT; n = 10, basal-OT, CCL19-OT and CCL21-OT) that were exposed to CCR7 ligands (1 µg/mL). (**B**) Migration index of T-cells from untreated patients (n = 7), pre-incubated for 3 h with increasing concentrations of ibrutinib (0/DMSO, 0.01, 0.1, 1, 10 µM) before adding CCR7 ligands (1 µg/mL). (**C**) Migration index of T-cells from treatment-naïve patients (n = 10), incubated for 3 h with ibrutinib [0/DMSO, or 0.1 µM], or for 30 min with anti-CCR7 mAb CAP-100 (10 µg/mL), or with the combination of both compounds before adding CCL19 or CCL21 (1 µg/mL). (**D**) Proportion of T-cells from treatment-naïve patients (n = 5) that migrated towards CCR7 ligands (1 µg/mL) after being exposed for 24 h to ibrutinib (0/DMSO or 0.1 µM). (**E**) CLL T-cells viability after ibrutinib treatment in vitro. T-cells, freshly obtained from untreated CLL patients, were cultured with ibrutinib (0/DMSO, 0.01, 0.1, 1, 10 µM) for 24 h. Then, the proportion of viable cells was determined by gating on 7-AAD-negative T-cells (n = 5). In (**A**–**D**), spontaneous migration not mediated by a chemotactic stimulus, was considered as basal migration. In (**A**–**E**), bars represent mean ± standard error of the mean (SEM). ns, not significant; *, *p* < 0.05; **, *p* < 0.01; ***, *p* < 0.001.

CLL T-cells from treatment-naïve patients show a markedly lower migratory capability towards CCR7 ligands than normal counterparts [30]. We therefore asked whether ibrutinib treatment might possibly restore impaired T-cell migration. To this end, we conducted comparative analyses of the in vitro CCR7 migratory response of T-cells from treatment-naïve versus ibrutinib OT CLL patients (Figure 2A). T-cells from both groups of patients showed similar migration indices, thus indicating that the inhibitor was not affecting CCR7-driven chemotaxis. To corroborate these findings, we incubated T-cells from treatment-naïve patients with different concentrations of ibrutinib before exposure to CCL19 or CCL21 (Figure 2B). Short incubation periods with ibrutinib did not modify migratory response, whereas it was drastically reduced with the novel CCR7-specific therapeutic antibody CAP-100, used as a bench reference for CCR7 inhibition (Figure 2C). In order to discard a lack of effect due to a suboptimal exposure to ibrutinib, we pre-incubated treatment-naïve CLL T-cells with 0.1 µM ibrutinib for 24 h before testing chemotaxis (Figure 2D). Again, migration indices were comparable to controls. Finally, we discarded an effect on cell viability after a long-term incubation period (Figure 2E), thus confirming previous results where no influence of ibrutinib on T-cell survival was observed [34]. Together, these findings confirmed that in vivo or in vitro treatment with ibrutinib was not modulating the CCR7-mediated migration of CLL T-cells. 

### 3.3. Ibrutinib Does Not Affect SLO Homing of T-Cells or Their Interstitial Motility within the LN Parenchyma

**Figure 3:**
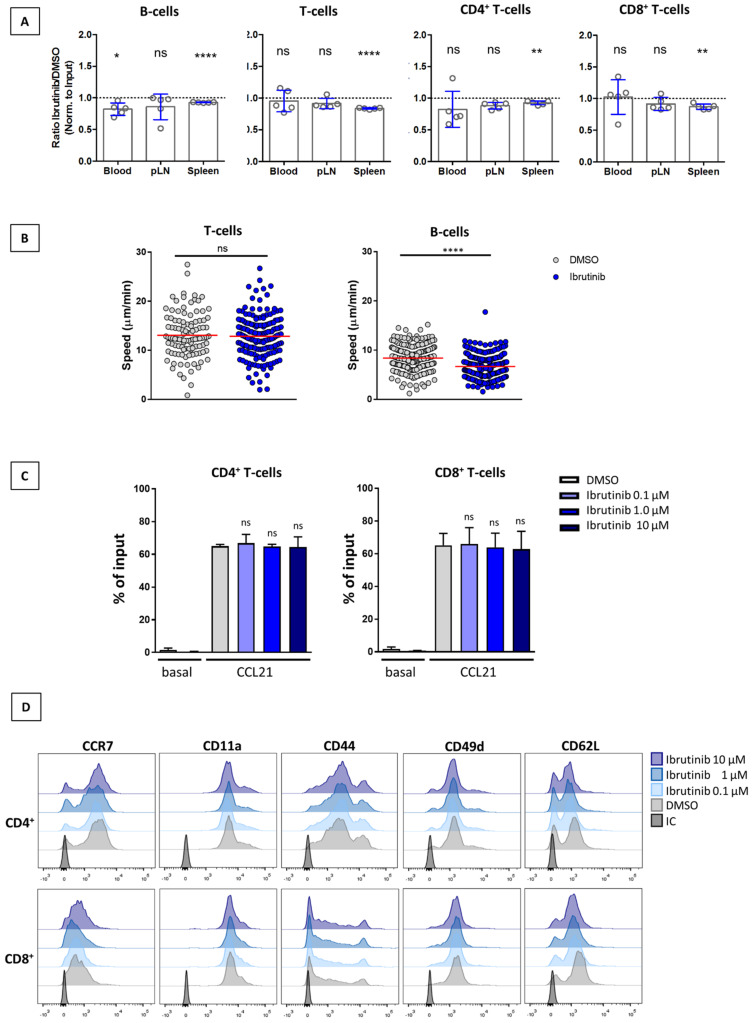
Ibrutinib does not alter the CCR7-mediated homing, motility or migration of murine T-cells. (**A**) In vivo homing of lymphocytes treated with ibrutinib. Fluorescently labeled cells were treated with either ibrutinib (1 µM) or DMSO and transferred in a 1:1 ratio into recipients (n = 5). The graph shows the ratio of ibrutinib/DMSO cells recovered from peripheral blood (PB), peripheral lymph nodes (pLN) and spleen 1 h post-adoptive transfer. Data were normalized to input and analyzed by a one-sample *t*-test against the theoretical value of “1” (equal recovery). (**B**) 2PM analysis on interstitial migration of T-cells and B-cells in the popliteal LN from DMSO or ibrutinib treated mice. The graphs show that mean T and B-cells track speeds in mice treated with 6 mg/kg ibrutinib (blue dots) or its vehicle (grey dots). Data were pooled from at least three mice/condition, from two independent experiments. (**C**) Treatment with ibrutinib does not modulate migration mediated by CCR7 in T-cells. Analysis of the CCL21-mediated chemotaxis in DMSO or ibrutinib pre-treated murine T-cells. The figure shows the proportion of migrating cells (% of input) as mean ± SD (n = 3). ns, not significant. (**D**) Ibrutinib does not change CCR7 or adhesion receptors expression of murine T-cells. Lymphocytes were treated with either DMSO or increasing concentrations of ibrutinib before staining them with a panel of mAbs directed against CCR7 or the following adhesion molecules: CD11a (LFA-1), CD44, CD49d (VLA-4), or CD62L. Histograms (representative examples) from CD4^+^ and CD8^+^ T-cells analysis are shown. ns, not significant; *, *p* < 0.05; **, *p* <0.01; ****; *p* < 0.0001.

Recently, Thelen et al. reported that genetic deletion of ITK impaired the CCR7-driven LN homing and interstitial migration within this organ of CD4^+^ T-cells [27]. Given these facts, we aimed to determine whether ibrutinib affected the homeostatic motility of T-cells towards and within nodal tissue. To this end, we performed in vivo homing assays with lymphocytes pre-treated with either ibrutinib (1 µM) or DMSO. The expected results must have resembled those by Thelen et al., who showed reduction in short-term recoveries of ~30% in spleen and ~50% in other SLO, while these cells were more abundant in PB (~25%). However, ibrutinib-treated T-cells were found in the LN of recipient mice to the same extent as untreated cells (Figure 3A) whereas B-cells showed a mild though not significant reduction of ~14% in the ratio ibrutinib/DMSO cells recovered from LN (B-cells: 0.86 ± 0.20 SD vs the theoretical value of “1” that indicates equal recovery). As for the spleens, all of the study subsets showed a minor (<10%) but consistent reduction in the number of recovered cells. Since the latter changes in SLO did not translate into a concomitant increase of cells in PB, we can exclude an effect of ibrutinib on homing. 

In order to study whether systemic ibrutinib treatment was able to reduce in vivo T-cell motility, we next performed intravital 2PM imaging on the popliteal LN of mice. In these settings, adoptively transferred, fluorescently labelled T- and B-cells (the latter used as controls) were allowed to recirculate for 48 h. Over the following three days, mice were treated daily with i.p. 6 mg/kg ibrutinib or vehicle (DMSO) [25]. As shown in Figure 3B and Supplementary Video S1, no effect of ibrutinib on T-cells was observed, while a significant decrease in the migration speed of B-cells within the parenchyma of the LNs was seen. To further confirm the lack of effect of ibrutinib in migratory responses, we conducted in vitro chemotactic assays using murine T-cells that were pre-exposed to different doses of ibrutinib (Figure 3C). When compared to control cells, CCL21-driven T-cell chemotaxis remained unaffected in CD4^+^ or CD8^+^ T-cells pre-treated with ibrutinib. Moreover, ibrutinib did not change the expression profile of CCR7 or adhesion receptors directly involved in CCR7-induced migration or homing, such as CD49d, CD11a, CD44, or CD62L [35,36,37] (Figure 3D). Overall, these findings confirmed that ibrutinib was not modifying CCR7-mediated migration in murine T-cells, either in a positive or negative manner.

## 4. Discussion

Ibrutinib treatment exhibits profound effects on the immunocompromised T-cell compartment in CLL patients, among which the T-cell lymphocytosis stands out [1,18,19,38]. Theoretically, this effect might be a consequence of either an impaired trafficking to SLO or an increased egress from these tissues, although the exact mechanisms underlying this homeostatic change have only been partially tackled. In this regard, Long et al. recently showed in a syngeneic CLL mouse model that ibrutinib was not enhancing egress of CLL T-cells, but rather their expansion and survival [14]. Nonetheless, clues on the other part of the equation, namely the entry of CLL T-cells in the LN, were not provided. For this reason, we focused on CCR7, the fundamental chemokine receptor needed for an effective PB-SLO recirculation of T-cells. Our results confirm that CCR7 migration is not modulated by ibrutinib in vivo or in vitro treatments, further suggesting that T-cells trafficking to LN/spleen may be preserved in CLL patients, thus supporting the expansion hypothesis by Long et al. [14]. A recent study by Thelen et al. shows that ITK signaling in T-cells, one of the best documented off-target cascades of ibrutinib, has a minor contribution to CCR7-induced motility in vitro and scanning behavior in vivo, while it seems to participate in the homing of naïve T-cells to SLO [27]. Interestingly, our homing assays with ibrutinib-treated cells were performed in similar settings but did not reproduce the findings by Thelen et al. Similarly, while intravital imaging confirmed that in vivo T cell motility in the LN parenchyma is reduced in the absence of ITK [27], our model showed no impact of ibrutinib on the same process. Together, these results suggest that phenotypes in ibrutinib-treated CLL patients do not necessarily reproduce that seen in knock-out mice. This finding is not strange when taking into account that several signaling pathways concurrently orchestrate migration towards CCR7 ligands in CLL [21,23]; therefore, a partial effect caused by ibrutinib of some of these proteins might be compensated by the others. Moreover, the fact that we found no differences in T-cell migration between naïve or ibrutinib-treated CLL patients strongly indicates that the BTK inhibitor is not improving the partially impaired basal migration of T-cells towards CCR7 ligands [30]. 

Despite the fact that ibrutinib does not seem to modulate CCR7 function, we cannot ignore the possibility that this inhibitor may weaken chemokine- or TCR-promoted adhesion capacities of CLL T-cells as it actually does in malignant CLL B-cells [11,32,39,40]. Although we have not specifically addressed these questions, our data on interstitial migration within the LN shows that these events are not happening in T-cells while they are in B-cells. Moreover, our flow cytometry data on CD4^+^ and CD8^+^ mouse T-cells confirm that ibrutinib does not change expression profiles of adhesion receptors directly involved in CCR7-induced migration or homing, such as CD49d, CD11a, CD44, or CD62L [35,36,37]. 

Notably, ibrutinib did not alter the expression of CCR7 in CLL CD3^+^ T-cells from OT or relapsed/refractory patients when compared to that observed in naïve patients. This result not only precludes ibrutinib-induced CCR7 down-modulation as a mechanism to regulate CCR7-driven homeostasis, but also contrasts with the reported ibrutinib-induced reduction of CCR7 in CLL B-cells [11,22,32,33]. Disparities between both compartments may be explained by different needs of the pro-apoptotic protein p66Shc, the expression of which is known to be selectively re-activated by ibrutinib in CLL cells, thus restoring physiological CCR7 levels and B-cell homeostasis [33,41,42]. 

Cell surface CCR7 facilitates T-cell categorization into two main groups [43]: CCR7^+^ T-cells, comprising naïve (T_N_) and central memory (T_CM_) T-cells, and CCR7^−^ T-cells, including effector memory (T_EM_), terminal effector (T_TE_) and tissue-resident memory (T_RM_) T-cells. It has been shown that ibrutinib increases CD4^+^ and CD8^+^ T-cell absolute numbers, mostly effector and effector memory T-cells [14,44]. Although our results confirm that the proportion of CCR7^+^ vs CCR7^−^ CLL T-cells remains stable in the PB of the three patient groups, our analysis did not include markers to distinguish different subsets. Nonetheless, several studies have reported stable or only modestly changed absolute and relative numbers of naïve and effector/memory CD8^+^ and CD4^+^ T-cells at several time points examined during ibrutinib treatment, even after several cycles of it [14,16,38,44,45,46]. 

Finally, the fact that ibrutinib clearly expands TCR repertoire diversity in CLL patients [46] without modifying CCR7-driven T-cell migration or interstitial migration, strongly suggests bystander mechanisms fostering ibrutinib-triggered T-cell-mediated immune responses. In this regard, ibrutinib favored the maturation and activation status of LPS-stimulated dendritic cells (DC), the up-regulating of CCR7 expression and the promoting of subsequent T-cell activation and proliferation [47]. Therefore, it is plausible that ibrutinib may cause a substantial increase in the likelihood of T-cell/DC encounters by changing homing input and/or scanning behavior in the latter antigen-presenting subset.

## 5. Conclusions

There are multiple aspects in the physiology of CLL T-cells that are modulated by ibrutinib. In this work we studied whether the drug-associated increase of circulating T-cell numbers reflected a forced retention in PB derived from the impaired functionality of CCR7. We showed that ibrutinib has no apparent effect on CCR7-mediated T-cell chemotaxis, homing to LN or spleen, and interstitial migration. In addition, the inhibitor did not modify surface CCR7 expression profiles in T-cells from CLL patients on treatment with ibrutinib, nor in patients who had developed refractory/relapsed disease to the drug. These results, gathered with those discarding the augmented release of CLL T-cells from the SLOs as a root for T-cell lymphocytosis, strongly support ibrutinib-mediated T-cell expansion as the most plausible cause for this physiological event.

## Data Availability

The datasets used and/or analyzed during the current study are available from the corresponding author on reasonable request.

## Supplementary Materials

The following supporting information can be downloaded at: https://www.mdpi.com/article/10.3390/cancers14112729/s1, Video S1: Nodal interstitial migration in mice treated with ibrutinib. Representative examples of lymphocytes migrating in the parenchyma of popliteal LNs of C57BL/6J mice treated with DMSO or ibrutinib 6 mg/kg. T cells are shown in red, B cells in green and HEVs in grey.
